# Effects of Surgery and Chemotherapy on Metastatic Progression of Prostate Cancer: Evidence from the Natural History of the Disease Reconstructed through Mathematical Modeling

**DOI:** 10.3390/cancers3033632

**Published:** 2011-09-20

**Authors:** Leonid Hanin, Marco Zaider

**Affiliations:** 1 Department of Mathematics, Idaho State University, 921 S. 8^th^ Avenue, Stop 8085, Pocatello, ID 83209, USA; 2 Center for Bioinformatics and Computational Genomics, Georgia Institute of Technology, 313 Ferst Drive, Suite 2127, Atlanta, GA 30332, USA; 3 Department of Medical Physics, Memorial Sloan-Kettering Cancer Center, 1275 York Avenue, New York, NY 10021, USA; E-Mail: zaiderm@mskcc.org

**Keywords:** cancer dormancy, cancer stem cell, chemotherapy, mathematical model, metastasis latency, metastatic progression, primary tumor, prostate cancer, radiotherapy, surgery

## Abstract

This article brings mathematical modeling to bear on the reconstruction of the natural history of prostate cancer and assessment of the effects of treatment on metastatic progression. We present a comprehensive, entirely mechanistic mathematical model of cancer progression accounting for primary tumor latency, shedding of metastases, their dormancy and growth at secondary sites. Parameters of the model were estimated from the following data collected from 12 prostate cancer patients: (1) age and volume of the primary tumor at presentation; and (2) volumes of detectable bone metastases surveyed at a later time. This allowed us to estimate, for each patient, the age at cancer onset and inception of the first metastasis, the expected metastasis latency time and the rates of growth of the primary tumor and metastases before and after the start of treatment. We found that for all patients: (1) inception of the *first* metastasis occurred when the primary tumor was undetectable; (2) inception of all or most of the surveyed metastases occurred before the start of treatment; (3) the rate of metastasis shedding is essentially constant in time regardless of the size of the primary tumor and so it is only marginally affected by treatment; and most importantly, (4) surgery, chemotherapy and possibly radiation bring about a dramatic increase (by dozens or hundred times for most patients) in the average rate of growth of metastases. Our analysis supports the notion of metastasis dormancy and the existence of prostate cancer stem cells. The model is applicable to all metastatic solid cancers, and our conclusions agree well with the results of a similar analysis based on a simpler model applied to a case of metastatic breast cancer.

## 1. Introduction

### 1.1. Two Paradigms of Cancer

According to the conventional paradigm, cancer emerges when one or a few adjacent cells acquire a number of irreversible oncogenic mutations which eventually perturb cell cycle controls and apoptotic regulation. Subsequent proliferation of the initial transformed cell(s) results in a malignant tumor that develops a capillary network and acquires the ability to invade surrounding tissues and metastasize. Within this framework, cancer is viewed as an alien entity that progresses sequentially through stages characterized by the extent of its anatomic spread – local, regional and distant. Metastases are considered independently growing tumors that arise from malignant cells shed by the primary tumor and seeded at various secondary sites. An upshot of this view is that cancer treatment should consist of eliminating all cancer cells and the earlier and more aggressive the treatment of the primary tumor the better the prognosis.

An alternative paradigm of cancer has recently started to crystallize on the basis of more than 100 years of extensive clinical observations, epidemiological studies and animal experiments (see [1-4] for a comprehensive review). According to this new paradigm, a malignant tumor is an organ-like entity that exists in a dynamic state of homeostasis with its microenvironment which can act either as a promoter or suppressor of cancer progression. An important aspect of such a homeostasis is the state of dormancy of small avascular primary and secondary tumors and solitary cancer cells. In particular, large numbers of cancer cells may circulate in the blood and lymph vessels. The state of dormancy is maintained through the balance between the factors of growth and angiogenesis, on the one hand, and inhibitors of these processes, on the other. Seeding of metastases may occur long before primary cancer manifests clinically, which makes cancer a systemic disease already at its very early subclinical stages. Primary and secondary tumors at various sites engage in a complex biochemical interaction that typically results in suppression of the growth of small tumors by larger tumors. Accordingly, resection of the primary tumor will weaken this inhibitory effect. Additionally, wound healing processes following resection of the primary may promote growth of secondary tumors and angiogenesis. This leads to disruption of the dormant state of metastases and causes their accelerated growth and vascularization. Therefore, surgery may not always be a good treatment option. Maintaining the state of homeostasis or dormancy may prove a better strategy of cancer control.

The goal of the present work is to confirm or confute, in the case of metastatic prostate cancer, the principal biomedical hypotheses that lie behind this alternative paradigm of cancer and to extend them to chemotherapy (see below). Three of these hypotheses are related to the natural history of the disease (group A) and the other three to the effects of surgery and chemotherapy on metastatic progression (group B). Our findings will show that, contrary to the commonly accepted views, these two modes of treatment of the primary tumor have only minor effect on the rate of metastasis shedding but have a dramatic accelerating effect on the rate of metastatic growth. We believe the same is true for radiotherapy. As yet another outcome, our analysis lends further support, if only indirect, to the notions of tumor dormancy and cancer stem cells.

Testing of the hypotheses will be accomplished through reconstruction of the individual natural history of cancer and the effects of its treatment on the basis of a comprehensive mathematical model of cancer progression developed in [5-7] and extended in this work. Parameters of the model are estimated from the data on volumes of primary tumor and metastases for a cohort of 12 prostate cancer patients diagnosed and treated at the Memorial Sloan-Kettering Cancer Center (MSKCC).

### 1.2. Working Hypotheses

A1. Metastatic dissemination off the primary tumor is an early event in the natural history of the disease that may occur long before primary tumor becomes clinically detectable.

A2. Prior to the start of irreversible proliferation in a secondary site, micrometastases or solitary cancer cells spend an extended period of time in a state of dormancy or free circulation.

A3. The primary tumor has a small subpopulation of “cancer stem cells” of relatively constant size characterized by self-renewal, capacity for fast proliferation and high metastatic potential.

B1. Treatment of the primary tumor has only limited effect on the process of metastasis shedding.

B2. Extirpation of the primary tumor by surgery may boost the proliferation of dormant or slowly growing metastases, trigger their vascularization and accelerate growth of vascular secondary tumors.

B3. Chemotherapy may also accelerate the growth of metastases, although not necessarily by the same mechanism.

These hypotheses are mainly focused on metastatic progression because metastases are responsible for about 90% of cancer-related deaths. Below we discuss the status of these hypotheses, briefly review supporting biomedical evidence and discuss underlying biological mechanisms. For a more extensive discussion, the reader is referred to the reviews [1-4].

### 1.3. Hypotheses—The Whys and Wherefores

A1. This hypothesis has been discussed in the medical literature for several decades [8-10]. It was estimated in [10] that more than 70% of cancer patients have occult metastases at presentation. Because hypothesis A1 deals with unobservable events, its direct confirmation may prove elusive. However, a wealth of indirect evidence points to its validity. For example, how else could one explain the fact that a significant fraction of patients whose primary tumor was diagnosed and removed at the earliest stages of cancer progression still develop distant metastases?

A2. The earliest report on circulating cancer cells goes back to 1869 [11] (see also [12]). Numerous modern studies of various types of cancer [13-16] showed that significant quantities of circulating tumor cells can be present in blood and lymph channels without clinically manifest metastases. The next critical step in the multi-stage process of metastasis formation is invading a host site and establishing there. High sensitivity of tumor cells to the conditions of host microenvironment and the resulting selective affinity of tumor cells to specific organs and tissues has been known since 1889 under the name of “seed and soil hypothesis” [17]. However, it was uncovered by subsequent pioneering studies [18, 19] that even if seeded into a fertile “soil,” a tumor cell may remain dormant in a secondary site yet retain its clonogenic capacity. Direct evidence of prevalent breast tumor cell dormancy was presented in [20]. The technique of *in vivo* video microscopy enabled direct observation and quantitative study of dormant cancer cells [21, 22]. Recently, tumor cell dormancy was recognized as a major new direction in cancer research [23]. It was found that about 1/3 of breast cancer patients 7–22 years after mastectomy and without any evidence of the disease had circulating tumor cells [24]. Their presence in peripheral blood was also confirmed in 24% of prostate cancer patients before prostatectomy [25] as well as in patients with undetectable PSA levels more than five years post-prostatectomy [25, 26].

A balance between proliferation, apoptosis and dormancy of cancer cells brings about the possibility that primary or secondary cancer remains subclinical for an extended period of time; as an example, breast cancer recurrence was reported to occur after 20 to 25 years of disease-free period [27].

The last critical step on the pathway leading to a detectable metastasis is induction of angiogenesis [28]. It was estimated that only about 4-10% of actively growing metastases eventually develop the capillary network which enables further growth [29].

The time period between shedding of a metastatic cell by the primary tumor and the beginning of its irreversible proliferation in a host site resulting in a clinically detectable secondary tumor will be referred to as *metastasis latency*. The latency period comprises the stages of free circulation, dormancy or slow avascular growth in a secondary site and induction of angiogenesis. In what follows, we will estimate the expected metastasis latency times for 12 prostate cancer patients.

A3. “Cancer stem cells” were first discovered in the case of acute myeloid leukemia [30]. Later their existence was confirmed for other types of hematologic cancers as well as for several solid cancers, including prostate cancer [31]. The hallmarks of cancer stem cells are self-renewal, pluripotency (*i.e.*, the ability to produce various types of cancer cells through differentiation), fast proliferation and high metastatic potential. The existence of cancer stem cells is highly consequential for cancer treatment, for it implies that targeting and eliminating, or at least controlling, a very small subpopulation of tumor cells is critical for the success of cancer therapy.

B1. Arguments in support of the hypothesis that local control may have only a limited effect on the probability and timing of distant failure have been so far mostly of three kinds: (a) assessing whether local and distant failure are statistically correlated events and whether the same clinical variables and risk factors predict for both of them; (b) observing various relationships between the age at local recurrence and distant failure (for example, patients who fail locally may display an increase in the hazard rate of the time from treatment to detection of metastases as well as larger number and volumes of metastases, as compared to locally-controlled patients); and (c) in the case of radiotherapy, examining the effects of dose escalation on cancer-specific survival. What these phenomenological considerations tend to neglect is heterogeneity of biological mechanisms underlying the effects of various modes of treatment of the primary tumor (such as surgery, external beam radiation, brachytherapy, and chemo- and hormonal therapy) on metastases. The probability and age at distant failure depend on four important characteristics of metastasis: (1) the rate of metastasis shedding by the primary tumor; (2) the fraction of metastases shed by the primary tumor that may potentially give rise to detectable secondary tumors in a given site; (3) the duration of metastatic latency; and (4) the site-specific rates of growth of metastases. The structure of the model employed in this work and the scarcity of data available for estimation of model parameters allowed us to study the effects of treatment of the primary on only two of these four characteristics – the rates of metastasis shedding (hypothesis B1) and growth (hypotheses B2 and B3). The model can be extended to include the effects of treatment of the primary on the duration of metastatic latency at the cost of introducing additional model parameters. However, parameter estimation for such an extended model would require much larger sample sizes or longitudinal data.

B2. Cancer patients who present even at late stages of the disease rarely have clinically manifest metastases; typically, they surface after the start of treatment. That this phenomenon is deeply rooted in basic cancer biology is postulated in hypothesis B2. This hypothesis addresses one of the multiple effects that a primary tumor exerts on other primary or secondary tumors. Experimental studies of these effects on animal models were conducted as early as the beginning of the 20^th^ century [32-35]. Numerous later works confirmed these early findings although changed their interpretation, see [2, 3] and references therein. The most important discovery within this realm of research is that larger tumors inhibit growth of smaller ones and, as a result, resection of large primary or secondary tumors accelerates the growth of smaller tumors. In particular, it was found that extirpation of the primary tumor triggers aggressive proliferation of dormant or slowly growing metastases and their vascularization [36-38]. This important finding was further supported by a number of clinical case studies including eight cases of non-seminomatous germ-cell testicular cancer [39] and three cases of melanoma [40, 41]. Additionally, and most importantly for the present study, substantial evidence of post-resection progression of metastatic disease in prostate cancer patients was presented in [42]. The validity of hypothesis B2 is supported by epidemiological analyses of the time course of post-surgery recurrence for various categories of cancer patients [43-45], propped by a simple mathematical model of breast cancer progression [46] and corroborated by an experimental study of accelerated growth of metastases after resection of the primary Lewis lung carcinoma in mice [47]. The hypothesis was also employed to explain the “mammography paradox”, *viz.* an unexpected increase in the mortality of pre-menopausal node-positive women aged 40-49 diagnosed with breast cancer as a result of screening-based early detection in a number of large-scale clinical trials [48-49]. The extent of accelerated growth of metastases was found to be proportional to the extent of surgery. As one example, tumor recurrence in non-metastatic colon cancer patients was markedly lower in a group that had laparoscopic surgery than for the open colectomy group [50]. Finally, even biopsy was reported to result in a measurable increase in the incidence of lung metastases in mice [51].

What is the mechanism of accelerated growth of metastases following resection (and possibly radiation treatment) of the primary tumor? Briefly, as hypothesized in [52] and confirmed in a host of other studies, many of which are reviewed in [1-4], the primary tumor and its microenvironment produce tumor growth factors as well as growth inhibitors. The growth factors are more easily degradable than growth inhibitors and propagate mostly by diffusion thus acting locally and promoting primary tumor growth. By contrast, growth inhibitors are more stable; as a result, they may reach remote secondary sites and impede the growth of metastases. The latter is also limited by various circulating angiogenesis inhibitors (such as angiostatin and endostatin). Removal of the primary tumor reduces production of growth inhibitors, which accelerates the growth of metastases. Additionally, and perhaps more importantly, healing of surgery- or radiation-related injury is accompanied by a surge in the local and systemic production of various growth and angiogenesis factors that act synergistically with the decrease in the levels of growth and angiogenesis inhibitors. This mechanism suggests that the same metastasis enhancing effect will also manifest for surgery or wounding unrelated to primary tumor. A statistical analysis based on 418 patients with advanced cancer reported in [53] confirmed that this indeed is the case. Similar but weaker effects may result even from biopsy [51]. The mechanisms described above seem to be also relevant to radiation therapy.

B3. A significant fraction of cancer patients treated with chemotherapeutic agents develops resistance to treatment. Undoubtedly, this effect is due to many mechanisms; one of them is selection of resistant cells in the target population. This process is mediated and amplified by the formation of spontaneous and chemotherapy-induced mutations, adaptive reactions of cancer cells causing them to evade cytotoxic action of drugs by switching to alternative metabolic and proliferative pathways, and removal of cytotoxic agents from cancer cells by transporting them across cell membrane due to the action of ATP-binding cassette transporters. Finally, chemotherapy, as any other treatment, confers survival advantage on faster proliferating cells, unless the latter are more sensitive to the treatment than slower growing cells. Selection of resistant and fast proliferating cells leads to the decreased efficiency and ultimate failure of chemotherapy.

Taken collectively, hypotheses A1, B2 and B3, if confirmed, would suggest that there is no such thing as “local treatment” *per se*: any intervention aimed at the primary affects metastatic progression. Therefore, in the present work, we will use the term “local treatment” only as an indication of intent rather than statement about the mechanism or outcome.

## 2. General Methodology

The above six hypotheses are formulated in terms of events and processes that are typically unobservable, or only partially observable, *in vivo*. In the present work, we bring mathematical modeling to bear on estimation of their probabilities, timing and rate characteristics. A distinct advantage of this approach is that mathematical models make it possible to relate unobservable quantities such as the age at disease onset or the start of its metastatic dissemination to clinical variables that are recorded months, years or even decades after the occurrence of the initiating micro-events.

The individual natural history of cancer consists of two important (but unobservable) pieces of information: (1) time to (or age at) critical micro-events such as the emergence of the first malignant clonogenic cell, shedding of metastases by the primary tumor, their seeding at various secondary sites and the start of their irreversible proliferation (termed *inception*); and (2) rate parameters descriptive of various cancer progression processes including growth of the primary tumor, shedding of metastases, and their seeding and growth at various secondary sites. The patient's observable clinical variables include age, stage and primary tumor volume at diagnosis, various biochemical or genomic markers, and clinical data resulting from follow-up studies such as the age at tumor recurrence or cancer related death and, for our purpose, site-specific number and volumes of detectable metastases. Establishing a quantitative relationship between parameters of the natural history of cancer and remote clinical endpoints and response variables is the apanage of mathematical modeling. Finally, because cancer progression involves substantial variability of the characteristics of primary and secondary tumors and their microenvironments and is impelled through a number of sporadically occurring random events [54], a stochastic approach would be the modeling tool of choice.

In this work, we apply a comprehensive stochastic model of cancer progression developed in [5-7] to metastatic prostate cancer and extend the mathematical formalism to the case where patients receive systemic treatment alone. The basic idea behind the model is to view the process of metastasis shedding by the primary tumor as a Poisson process whose intensity is proportional to a certain power of the size of the primary tumor [6,7], see also [5]. The model allows for arbitrary laws of growth of the primary tumor and metastases, and leads to an explicit formula for the conditional distribution of the volumes of detectable metastases in a certain secondary site, given their number, at any time point with an intact, excised or regrowing primary tumor [6]. The basic model developed in [6] was extended to accommodate distinct rates of growth of metastases prior to and after excision the primary tumor [7,55]. In the present work, we further extend it to incorporate two distinct laws of growth of the primary tumor: before and after the start of treatment. In the case of non-recurring, surgically removed primary tumor the model reduces to the one developed in [7]. A parametric version of the extended model based on exponential growth of the primary tumor and metastases, and exponentially distributed metastasis latency times is developed in Section 5. Knowledge of identifiable model parameters allows one to estimate the most important temporal and rate characteristics of the natural history of metastatic cancer and the effects of treatment. Surprisingly (and reassuringly), the model developed in the present work is sensitive enough to correctly predict whether a given patient had surgery.

The model designed in [7] was applied to a breast cancer patient who developed, by age 82 and 8 years after diagnosis and resection of the primary, 31 detectable bone metastases [55]. The model provided an excellent fit to the empirical distribution of the observed volumes of metastases and led to the following patient-specific conclusions [55]:

1. The onset of the disease occurred at age 42, about 32 years prior to primary diagnosis.
2. Inception of the first metastasis occurred at age 44.5, that is, about 29.5 years prior to the primary diagnosis and 2.5 years after the onset of the disease at which time the primary tumor was extremely small and certainly undetectable.
3. Inception of all detected metastases except one occurred before excision of the primary.
4. The expected metastasis latency time was about 79.5 years (which means that at the time of surveying most metastases were still dormant and undetectable).
5. Resection of the primary tumor was followed by a 32-fold increase in the rate of growth of bone metastases notwithstanding the fact that after surgery the patient was put on tamoxifen that suppresses growth of metastases and has anti-angiogenic effect.
6. The process of metastasis shedding was essentially homogeneous (*i.e.*, independent of the volume of the primary tumor), which suggests the presence within the tumor of a small self-renewing subpopulation of relatively constant size consisting of cells with high metastatic potential. This may serve as indirect evidence for the existence of breast cancer stem cells.

In what follows we examine the applicability of these conclusions to prostate cancer patients.

## 3. A Mathematical Model of the Individual Natural History of Metastatic Cancer

The processes of metastasis formation, growth and progression are complex, heterogeneous and selective [10,56,57]. To form a micro-metastasis in an organ or tissue, a malignant cell has to detach itself from the primary tumor, degrade extracellular matrix, intravasate, traverse the circulatory network, evade attacks by the immune system, extravasate, invade a secondary site, survive through the dormancy period, start to proliferate and induce angiogenesis. As a result of this multi-stage selection process, only a tiny fraction of cells shed by the primary tumor give rise to actively growing metastases [10,56,57].

**Figure 1.**
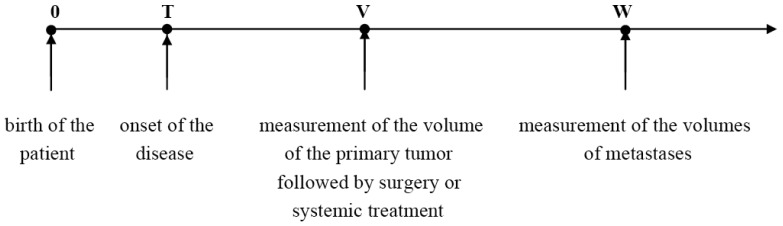
Timeline of the natural history and treatment of metastatic cancer.

The temporal natural history of metastatic cancer is commonly divided into three overlapping periods: disease-free period, primary tumor growth and metastatic progression. These periods and relevant model assumptions are described below and illustrated in Figure 1.

**Disease-free period** begins with the birth of an individual (or start of exposure to a carcinogen) and ends with the appearance of the first malignant clonogenic cell, a development termed the *onset of the disease*.

### Primary tumor dynamics

The size of the primary tumor (that is, the total number of tumor cells) at any time t counted from the age T of disease onset will be denoted by Φ(t). Prior to the start of treatment, the growth of the primary tumor is governed by a function Φ_0_ and thereafter by another function Φ_1_, which acts multiplicatively on the size of the primary tumor at the start of treatment (at age V). The function Φ_0_ is strictly increasing, continuous and satisfies the initial condition Φ_0_(0) = 1. As to the function Φ_1_, it is continuous but not necessarily increasing. In particular, for a non-recurrent excised tumor, Φ_1_ = 0. Functions Φ_0_ and Φ_1_ may depend on one or several parameters. We denote by φ the inverse function for Φ_0_. It follows from the above assumptions that

$$\Phi \left(\text{t}\right)=\{\begin{array}{c} \Phi_{0}\left(t\right),\\ \Phi_{1}\left(t-\left(\text{V}-T\right)\right)\Phi_{0}\left(V-T\right), \end{array}\begin{array}{c} \text{if}\;0\leq t\leq V-T\\ \text{if}\;t>V-T \end{array}$$

### Metastasis formation

The process of metastasis shedding is governed by a Poisson process with rate μ proportional to the number, N(t), of metastasis-producing cells at time t: μ(t) = α_0_N(t), where α_0_ > 0 is the rate of metastasis shedding per cell. Because N(t) is unobservable, we relate it to the primary tumor size Φ(t) through the formula N(t) = α_1_Φ^θ^ (t) with some constants α_1_ > 0 and θ ≥ 0. The value θ = 1 means that a constant fraction of cells in a tumor have metastatic potential. It is known that many solid tumors enclose a core of hypoxic, clonogenically sterile cells or even a broth of proteins, while actively proliferating clonogenic cells are concentrated near the tumor surface; in this case one would expect θ = 2/3. Finally, the case θ = 0 corresponds to the existence of a relatively stable, self-renewing subpopulation of metastasis-producing cells within the primary tumor. In summary, the rate of metastasis shedding is:

(1) $$\mu \left(\text{t}\right)=\alpha \;\Phi^{\theta }\left(\text{t}\right)$$

where α = α_0_ α_1_. In the case θ = 0 the rate of metastasis shedding μ is constant and the underlying Poisson process is homogeneous.

It is further assumed that metastases shed by the primary tumor give rise to clinically detectable secondary tumors in a given site independently of each other and with the same probability q. Therefore [58, pp. 257-259], inception of metastases in the site in question is governed by a Poisson process with intensity ν = q μ. Each viable metastasis is assumed to spend some random latency time between detachment from the primary tumor and its inception in a secondary site. We assume that latency times for different viable metastases are independent and identically distributed with some probability density function (pdf) f and corresponding cumulative distribution function (cdf) F. Then, see e.g. [59], the resulting process of metastasis inception is again a Poisson process with the rate

$$\lambda \left(\text{t}\right)=\int_{0}^{t}v\left(s\right)f\left(t-s\right)ds$$

### Timeline of the natural history of metastatic cancer and observables

Suppose that the observed primary tumor size at age V is S. Then the patient's age T at the disease onset is given by the formula

(2) T=V−φ(S)

We will assume that local or systemic treatment was given (or started) at age V, and that at age W, W > V, a certain number, n, of metastases were detected in the same secondary site with the observed volumes X_1_, X_2_,…, X_n_, where X_1_ < X_2_ < … < X_n_. Thus, 0 < T < V < W (Figure 1).

### Growth of metastases

Prior to the start of treatment, the growth of the size of any viable metastasis in a given secondary site is governed by a function Ψ_0_, while during or after the treatment, the size of the metastasis is growing according to a potentially different function Ψ_1_, which acts multiplicatively on the size of the metastasis at the start of treatment. We assume for simplicity that actively growing metastases start from a single cell. Functions Ψ_0_, Ψ_1_ are strictly increasing, differentiable, and satisfying the initial condition Ψ_0_(0) = Ψ_1_(0) = 1. Additionally, they may depend on one or several parameters. It follows from our assumptions that the size Ψ (y) of a viable metastasis at time y from inception is given by:

(3) $$\Psi \left(\text{y}\right)=\{\begin{array}{cc} \Psi_{1}\left(y\right), & \text{if}\;0\leq y\leq W-V\\ \Psi_{0}\left(y-\left(W-V\right)\right)\Psi_{1}\left(W-V\right), & \text{if}\;W-V<y\leq W-T \end{array}$$

This function is strictly increasing, continuous, piecewise differentiable and satisfies the condition Ψ(0) = 1.

### Secondary metastasis

Secondary metastasizing (that is, formation of “metastasis of metastasis”) to a given site, both from other sites and from within, is assumed negligible.

### Metastasis detection

The volume of a metastasis becomes measurable when it reaches some threshold value m. This value and the accuracy of volume measurement are determined by the sensitivity of imaging technology. In case of PET/CT imaging involved in this study, m = 0.5 cm^3^, and the accuracy of volume determination is one voxel, *i.e.*, approximately 0.065 cm^3^.

### Effects of treatment

Because the rate of secondary metastasizing is assumed negligible, the formation of new metastases is stopped at the time of resection of a non-recurrent primary tumor. Any mode of local or systemic treatment (surgery, radiation, chemo- or hormonal therapy) is assumed to affect metastases after their inception in a given secondary site only through the rate of their growth (and not through prolongation of their latency times).

## 4. Distribution of the Sizes of Detectable Metastases

Let X be the size of a detectable metastasis with inception time Y (relative to the onset of the disease) that was surveyed at age W. Then:

$$\text{X}=\Psi \left(\text{W}-\text{T}-\text{Y}\right)=\{\begin{array}{cc} \Psi_{0}\left(V-T-Y\right)\Psi_{1}\left(W-V\right), & \text{if}\;Y<V-T\\ \Psi_{1}\left(W-T-Y\right), & \text{if}\;Y\geq V-T \end{array}$$

where W – T – Y is the *metastasis progression time* from inception to detection and function Ψ is given by (3). The maximum value of X is:

(4) $$\text{M}=\Psi_{0}\left(V-T\right)\Psi_{1}\left(W-V\right),$$

and the inverse function, ψ:= Ψ^−1^, is given by:

(5) $$\psi \left(\text{x}\right)=\{\begin{array}{cc} {\Psi_{1}}^{-1}\left(x\right), & \text{if}\;1\leq x\leq \Psi_{1}\left(W-V\right)\\ {\Psi_{0}}^{-1}\left(\frac{x}{\Psi_{1}\left(W-V\right)}\right)+W-V, & \text{if}\;\Psi_{1}\left(W-V\right)<x\leq M \end{array}$$

The distribution of the sizes of metastases in a given secondary site is specified in the following theorem [6,7,55].

### Theorem 1

The sizes *X_1_ < X_2_ < … < X_n_* of metastases in a given secondary site that are detectable at age *W* are equidistributed, given their number *n*, with the vector of order statistics for a random sample of size *n* drawn from the distribution with the following pdf:

(6) $$p\left(x\right)=\omega \left(W-T-\psi \left(x\right)\right)\psi^{\prime }\left(x\right),\;m\leq x\leq M,$$

and *p(x)* = 0 for *x* ∉ *[m, M]*, where the tumor onset time *T* is given by (2), function *ψ* is defined in (5), *M* is specified in (4),

(7) $$\omega \left(t\right)=\frac{\int_{0}^{\min\left\{t,V-T\right\}}\Phi^{\theta }\left(s\right)f\left(t-s\right)ds}{\int_{0}^{\min\left\{W-T-\psi \left(m\right),V-T\right\}}\Phi^{\theta }\left(s\right)F\left(W-T-\psi \left(m\right)-s\right)ds},\;0\leq t\leq W-T-\psi \left(m\right)$$

and *F* is the cdf of the metastasis latency time corresponding to the pdf *f*.

For a proof of Theorem 1, see [6]. Notice that the distribution given by Equations (6)-(7) is free of parameters α, q and sample size n. Observe also that for θ = 0 we have:

(8) $$\omega \left(t\right)=\frac{F\left(t\right)-F\left(\max\left\{0,t-V+T\right\}\right)}{\int_{0}^{\min\left\{W-T-\psi \left(m\right),V-T\right\}}F\left(W-T-\psi \left(m\right)-s\right)ds},\;0\leq t\leq W-T-\psi \left(m\right)$$

In this case, the distribution p(x) is independent of the laws of primary tumor dynamics before and after the start of treatment. Setting in Equations (6)-(8) m = c, the volume of a single cancer cell, we obtain the distribution of the sizes of all (both occult and detectable) metastases in a given site. Finally, the site-specific total number of viable metastases at age t > T is Poisson distributed with parameter (expected value)

(9) $$\text{E}\left(\text{t}\right)=\text{q}\;\alpha \int_{0}^{t-T}\Phi^{\theta }\left(s\right)F\left(W-T-s\right)ds$$

while the probability of developing viable metastases at age t is 1-exp{-E(t)}. In particular, for a tumor resected at age V we have:

(10) $$\text{E}\left(\text{t}\right)=\text{q}\;\alpha \int_{0}^{\min\left\{t-T,V\right\}}\Phi^{\theta }\left(s\right)F\left(W-T-s\right)ds$$

Due to the non-stationarity of the process of metastasis seeding and the lack of a “natural” order for listing detectable metastases, the sizes (or volumes) of metastases detectable in a certain secondary site at a given time do not form a random sample from a probability distribution. However, it follows from Theorem 1 that the distribution of any rearrangement-invariant statistic based on observations X_1_, X_2_,…, X_n_ would be identical to the distribution of the same statistic based on a random sample of size n drawn from the pdf p given by Equations (6)-(7). In particular, the joint likelihood of the observations X_1_, X_2_,…, X_n_, where X_1_ < X_2_ < … < X_n_, given by the formula

$$\text{L}\left({\text{X}}_{1},{\text{X}}_{2},\ldots ,{\text{X}}_{\text{n}}\right)=\text{n}!\prod_{i=1}^{n}p\left(X_{i}\right)$$

has the same form (apart from the factor n!) it would take should the observations X_1_, X_2_, …, X_n_ form a random sample from the distribution with pdf p. Therefore, identifiable parameters of a suitably parameterized model of the natural history of metastatic cancer described in Section 3 can be estimated using the method of maximum likelihood.

## 5. Distribution of the Sizes of Detectable Metastases for Exponentially Growing Primary Tumor and Metastases

In this section, we introduce a parametric version of the general model of cancer natural history described in Section 3 and compute the distribution p(x) underlying the site-specific sizes of detectable metastases given by Equations (6)-(7).

Suppose that the size of the primary tumor grows exponentially with constant rate β_0_ > 0 before treatment and with rate β_1_ after the start of treatment: Φ_0_(t) = exp{β_0_t}, 0 ≤ t ≤ V - T, where time t is counted from the age T of tumor onset, and Φ_1_(t) = exp{β_1_t}, where time t is counted from the start of treatment. Note that rate β_1_ can be negative. Then for φ, the inverse function for Φ_0_, we have φ(s) = (ln S)/β_0_, so that T = V - (ln S)/β_0_, see Equation (2). Clearly, we must have T > 0, which implies

(11) $$\beta_{0}>\frac{\ln\;S}{V}$$

We will assume that before the start of treatment metastases in the site of interest grow exponentially with rate γ_0_ > 0, so that Ψ_0_(t) = exp{γ_0_t}. Note that for all 12 patients analyzed in this work their metastases reached considerable sizes at the time of surveying. That is why we are assuming that after the start of treatment the sizes of metastases also grow exponentially with rate γ_1_> 0. Then Ψ_1_(t) = exp{γ_1_t}, and Equation (3) becomes:

$$\Psi \left(\text{y}\right)=\{\begin{array}{cc} e^{\gamma_{1}y}, & \text{if}\;0\leq y\leq W-V\\ e^{\gamma_{0}y+\left(\gamma_{1}-\gamma_{0}\right)\left(W-V\right)}, & \text{if}\;W-V<y\leq W-T \end{array}$$

while for ψ = Ψ^−1^ Equation (5) yields:

$$\psi \left(\text{x}\right)=\{\begin{array}{cc} \frac{\ln\;x}{\gamma_{1}}, & \text{if}\;1\leq x\leq e^{\gamma_{1}\left(W-V\right)}\\ \frac{\ln\;x}{\gamma_{0}}+\left(1-\frac{\gamma_{1}}{\gamma_{0}}\right)\left(W-V\right), & \text{if}\;e^{\gamma_{1}\left(W-V\right)}<x\leq e^{\gamma_{0}\left(V-T\right)+\gamma_{1}\left(W-V\right)} \end{array}$$

Suppose additionally that metastasis latency times are exponentially distributed with the expected value ρ: f(s) = ρ^−1^e^−s/ρ^, s > 0.

The resulting parametric model of cancer natural history depends on the following 8 parameters: β_0_ (the rate of growth of the primary tumor prior to treatment), β_1_ (the rate of growth of the primary tumor after the start of treatment), α and θ (two parameters involved in the expression (1) for the rate of metastasis shedding), q (the probability that a metastases shed by the primary tumor will evolve into a viable, potentially detectable secondary tumor), γ_0_ (the rate of growth of metastases in the presence of untreated primary tumor), γ_1_ (the rate of growth of metastases after the start of treatment) and ρ (the mean metastasis latency time). Recall, however, that the distribution of the site-specific sizes of metastases depends only on 6 parameters: β_0_, β_1_, θ, γ_0_, γ_1_ and ρ.

Introduce the following alternative set of 6 model parameters:

$$\text{A}=\exp\left\{\gamma_{1}\left(W-V\right)\right\},\text{M}=\exp\left\{\gamma_{0}\left(V-T\right)+\gamma_{1}\left(W-V\right)\right\},$$

(12) $${\text{a}}_{0}=\frac{\beta_{0}\theta }{\gamma_{0}},{\text{a}}_{1}=\frac{\beta_{1}\theta }{\gamma_{1}},{\text{b}}_{0}=\frac{1}{\gamma_{0}\rho },{\text{b}}_{1}=\frac{1}{\gamma_{1}\rho }$$

Note that 0 < A < M.

### 5.1. The Case of Surgery with Non-Recurrent Primary Tumor

The case where the primary tumor was resected at age V and did not recur by age W was considered in [7,55]. It arises from a more general model described in Section 3 if one sets Φ_1_ = 0. Accordingly, the corresponding 5-parameteric version of the general 6-parametric parametric model obtains by setting β1 → − ∞. The pdf, p(x), underlying the site-specific distribution of the sizes of metastases at age W is given by the following expressions computed on the basis of Equations (6)-(7) [7]:

1. If *A* ≤ *m*, then
  (13) $$p\left(x\right)={\left(C_{1}x\right)}^{-1}\left[{\left(\frac{M}{x}\right)}^{a_{0}}-{\left(\frac{x}{M}\right)}^{b_{0}}\right],\;m\leq x\leq M,$$
  where
  (14) $$C_{1}={a_{0}}^{-1}\left[{\left(\frac{M}{m}\right)}^{a_{0}}-1\right]-{b_{0}}^{-1}\left[1-{\left(\frac{m}{M}\right)}^{b_{0}}\right].$$
2. If *A* > *m*, then
  (15) $$p\left(x\right)=\{\begin{array}{cc} \frac{b_{1}}{b_{0}}{\left(C_{2}x\right)}^{-1}\left[{\left(\frac{M}{A}\right)}^{a_{0}}-{\left(\frac{A}{M}\right)}^{b_{0}}\right]{\left(\frac{x}{A}\right)}^{b_{1}}, & m\leq x\leq A\\ {\left(C_{2}x\right)}^{-1}\left[{\left(\frac{M}{x}\right)}^{a_{0}}-{\left(\frac{x}{M}\right)}^{b_{0}}\right], & A\leq x\leq M \end{array}$$
  where
  (16) $${\text{C}}_{2}={b_{0}}^{-1}\left[{\left(\frac{M}{A}\right)}^{a_{0}}-{\left(\frac{A}{M}\right)}^{b_{0}}\right]\;\left[1-{\left(\frac{m}{A}\right)}^{b_{1}}\right]+{a_{0}}^{-1}\left[{\left(\frac{M}{A}\right)}^{a_{0}}-1\right]-{b_{0}}^{-1}\left[1-{\left(\frac{A}{M}\right)}^{b_{0}}\right]$$

Recall also that p(x) = 0 for x < m or x > M. The model (13)-(16) will be called the *Surgery model*.

We will also consider a limiting case of the above parametric model where θ = 0. Here the Poisson process of metastasis shedding is homogeneous. Accordingly, this model will be termed the *Homogeneous model*. Letting a_0_ → 0 in the Surgery model (13)-(16) we have:

1. If *A* ≤ *m*, then
  (17) $$p\left(x\right)={\left(C_{1}x\right)}^{-1}\left[1-{\left(\frac{x}{M}\right)}^{b_{0}}\right],\;m\leq x\leq M,$$
  where
  (18) $$C_{1}=\ln\frac{M}{m}-{b_{0}}^{-1}\left[1-{\left(\frac{m}{M}\right)}^{b_{0}}\right].$$
2. If *A* > *m*, then
  (19) $$p\left(x\right)=\{\begin{array}{cc} \frac{b_{1}}{b_{0}}{\left(C_{2}x\right)}^{-1}\left[1-{\left(\frac{A}{M}\right)}^{b_{0}}\right]\;{\left(\frac{x}{A}\right)}^{b_{1}}, & m\leq x<A\\ {\left(C_{2}x\right)}^{-1}\left[1-{\left(\frac{x}{M}\right)}^{b_{0}}\right], & A\leq x\leq M \end{array}$$
  where
  (20) $$C_{2}=\ln\frac{M}{A}+{b_{0}}^{-1}\left[1-{\left(\frac{A}{M}\right)}^{b_{0}}\right]\;\left[1-{\left(\frac{m}{A}\right)}^{b_{1}}\right]-{b_{0}}^{-1}\left[1-{\left(\frac{A}{M}\right)}^{b_{0}}\right]$$

The Surgery model (13)-(16) depends on 5 parameters A, M, a_0_, b_0_, b_1_, while its homogeneous version (17)-(20) depends on 4 parameters A, M, b_0_, b_1_. As shown in [7], in the case A > m all parameters of both models are identifiable (for an at length discussion of identifiability of stochastic models, see [60]).

### 5.2. The Case of Non-Resected or Resected Recurrent Tumor

In this case the distribution p(x) obtained from Equations (6)-(7) has the following form.

1. If *A* ≤ *m*, then Equations (13)-(14) apply.
2. If *A > m*, then
  (21) $$p\left(x\right)=\{\begin{array}{cc} {\left(C_{2}x\right)}^{-1}\frac{b_{1}}{b_{0}}\left\{\left[{\left(\frac{M}{A}\right)}^{a_{0}}-{\left(\frac{A}{M}\right)}^{b_{0}}\right]{\left(\frac{x}{A}\right)}^{b_{1}}+\frac{b_{1}}{b_{0}}\frac{a_{0}+b_{0}}{a_{1}+b_{1}}{\left(\frac{M}{A}\right)}^{a_{0}}\left[{\left(\frac{A}{x}\right)}^{a_{1}}-{\left(\frac{x}{A}\right)}^{b_{1}}\right]\right\}, & m\leq x<A\\ {\left(C_{2}x\right)}^{-1}\left[{\left(\frac{M}{x}\right)}^{a_{0}}-{\left(\frac{x}{M}\right)}^{b_{0}}\right], & A\leq x\leq M \end{array}$$
  where
  (22) $${\text{C}}_{2}={a_{0}}^{-1}\left[{\left(\frac{M}{A}\right)}^{a_{0}}-1\right]+{b_{0}}^{-1}\left\{{\left(\frac{M}{A}\right)}^{a_{0}}\left[1-{\left(\frac{m}{A}\right)}^{b_{1}}\right]+{\left(\frac{A}{M}\right)}^{b_{0}}{\left(\frac{m}{A}\right)}^{b_{1}}-1\right\}+{\left(\frac{b_{1}}{b_{0}}\right)}^{2}\frac{a_{0}+b_{0}}{a_{1}+b_{1}}{\left(\frac{M}{A}\right)}^{a_{0}}\left\{{a_{1}}^{-1}\left[{\left(\frac{A}{m}\right)}^{a_{1}}-1\right]-{b_{1}}^{-1}\left[1-{\left(\frac{m}{A}\right)}^{b_{1}}\right]\right\}$$

The corresponding Homogeneous model (θ = 0) obtains from the above *Full model* by letting a_0_, a_1_→ 0:

1. If *A* ≤ *m*, then Equations (17)-(18) apply.
2. If *A* > *m*, then
  (23) $$p\left(x\right)=\{\begin{array}{cc} {\left(C_{2}x\right)}^{-1}\frac{b_{1}}{b_{0}}\left[1-{\left(\frac{A}{M}\right)}^{b_{0}}{\left(\frac{x}{A}\right)}^{b_{1}}\right], & m\leq x\leq A\\ {\left(C_{2}x\right)}^{-1}\left[1-{\left(\frac{x}{M}\right)}^{b_{0}}\right], & A\leq x\leq M \end{array}$$
  with
  (24) $${\text{C}}_{2}=\ln\frac{M}{A}+\frac{b_{1}}{b_{0}}\ln\frac{A}{m}-{b_{0}}^{-1}\left[1-{\left(\frac{A}{M}\right)}^{b_{0}}{\left(\frac{m}{A}\right)}^{b_{1}}\right]$$

The Full model (13), (14), (21), (22) depends on six parameters A, M, a_0_, a_1_, b_0_, b_1_, while its Homogeneous version (17), (18), (23), (24) depends on four parameters A, M, b_0_, b_1_. An argument similar to the one developed in [7] would show that in the case A > m all parameters of both models are jointly identifiable. Note also that in the case where treatment affects the rate of growth of metastases (γ_1_ ≠ γ_0_) function p(x) is discontinuous at point A and p(A+)/p(A-) = γ_0_/γ_1_.

In what follows, quantities x, m, A and M will be expressed as volumes assuming the average volume, c, of a cancer cell to be 10^−9^ cm^3^. Observe, however, that because the function xp(x) in all cases depends only on the *ratios* of metastasis sizes x, m, A and M, the likelihood maximizing parameters are independent of c.

## 6. Computing Biological Parameters

The Full 6-parametric model of the natural history of cancer is determined by the biological parameters β_0_, β_1_, θ, γ_0_, γ_1_ and ρ. Equations (12) enable their expression through model parameters A, M, a_0_, a_1_, b_0_, b_1_. First, observe that

$$\gamma_{1}=\frac{\ln\;A}{W-V},\;\gamma_{0}=\frac{b_{1}\ln\;A}{b_{0}\left(W-V\right)}\;\text{and}\;\rho =\frac{W-V}{b_{1}\;\ln\;A}$$

Next, the expression for parameter M allows us to compute the disease onset time:

(25) $$\text{T}=\text{V}-\frac{1}{\gamma_{0}}\ln\frac{M}{A}=\text{V}-\frac{b_{0}\left(W-V\right)}{b_{1}\ln A}\ln\frac{M}{A}$$

In view of the inequality T > 0, model parameters should satisfy the following constraint:

(26) $$\frac{b_{0}}{b_{1}}=\frac{\gamma_{1}}{\gamma_{0}}<\frac{V\ln A}{\left(W-V\right)\ln\frac{\text{M}}{\text{A}}}$$

Computing the other three biological parameters requires the knowledge of the primary tumor size, S, at age V:

(27) $$\beta_{0}=\frac{b_{1}\ln\;A\;\ln\;S}{b_{0}\left(W-V\right)\ln\frac{\text{M}}{\text{A}}},\;\beta_{1}=\frac{a_{1}}{a_{0}}\frac{\ln\;A\;\ln\;S}{\left(W-V\right)\ln\frac{\text{M}}{\text{A}}}\;\text{and}\;\theta =a_{0}\frac{\ln\frac{\text{M}}{\text{A}}}{\ln\;S}$$

Because the volume, S_v_, of the primary tumor was estimated by pathologists based on a rough estimate of tumor margins, determination of the primary tumor size S = S_v_/c, where c is the volume of a single cancer cell, involves a considerable error. Yet another source of error is our assumption that c=10^−9^ cm^3^. Furthermore, for eight patients the data on the volume of the primary was unavailable and was ascribed a value of 20 cm^3^, see Table 1. However, these errors result in only minor deviation in the values of biological parameters β_0_, β_1_, θ due to the fact that S appears in the formulas for these parameters under the sign of logarithm. Note that parameters γ_0_, γ_1_, ρ and the age at disease onset T are independent of S.

## 7. The Data Set

To estimate model parameters, we used a data base of prostate cancer patients diagnosed and treated at MSKCC. To be useful for our analysis, the patients had to satisfy the following requirements: (1) availability of whole body PET/CT scans; (2) the number of metastases in a single secondary site (e.g., the skeletal system) is large enough (≥10); and (3) W > V, where V is the age at which the volume of the primary was measured immediately prior to surgery and/or start of systemic treatment while W is the age at metastasis surveying. Only 12 patients in the data base satisfied these conditions. Information on their gross primary tumor volume was generally unavailable and, when obtainable, quite likely affected by substantial errors. Among the 12 patients, one had surgery (radical prostatectomy) and one received surgery and external beam radiotherapy. Additionally, all 12 patients were given complex combinations and time courses of chemotherapy and adjuvant hormonal therapy. Relevant clinical variables for the 12 patients are given in Table 1. The number, n, of bone metastases detected in these patients varied between 10 and 58. When applying the above model of cancer progression we assumed that parameters β_1_ and γ_1_ represented the average rates of growth of the primary tumor and bone metastases, respectively, over the entire period from the start of treatment (age V) to metastasis survey (age W).

## 8. Results

Parameters A, M, a_0_, a_1_, b_0_, b_1_ of the Full model were estimated by maximizing the likelihood function L under constraints (11) and (26). In all cases, the optimal value of parameter A satisfied the condition A > m which prevented the model from degeneration, see Section 5. Discontinuity of the likelihood as function of A at the observed volumes of metastases and the presence of multiple local maxima prohibited the use of gradient methods for likelihood maximization. Instead, we used a genetic global optimization algorithm (Differential Evolution) built into Mathematica package.

**Figure 2.**
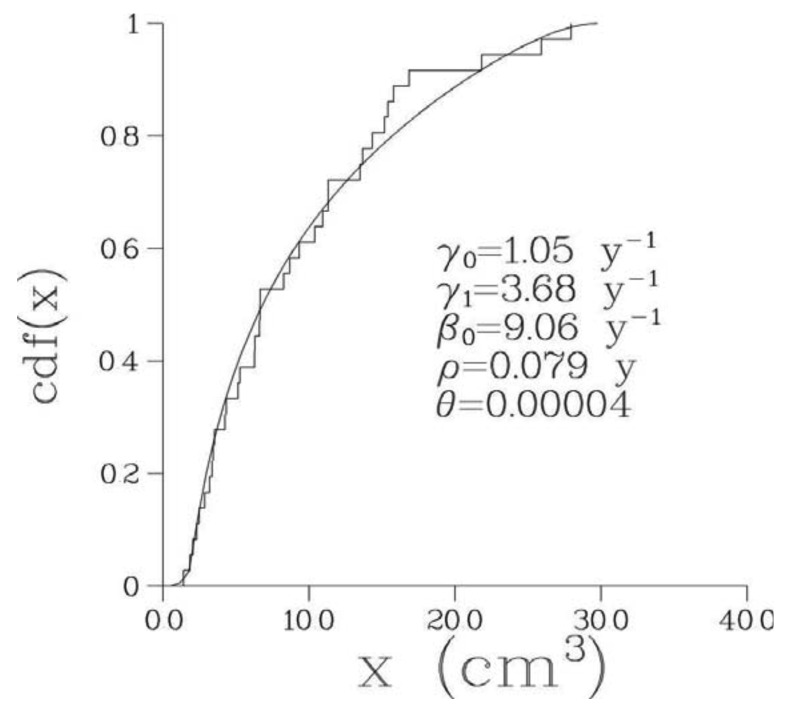
Empirical (stepwise curve) and theoretical (continuous curve) model based cumulative distribution functions for the volumes of detectable bone metastases for patient 1.

**Figure 3.**
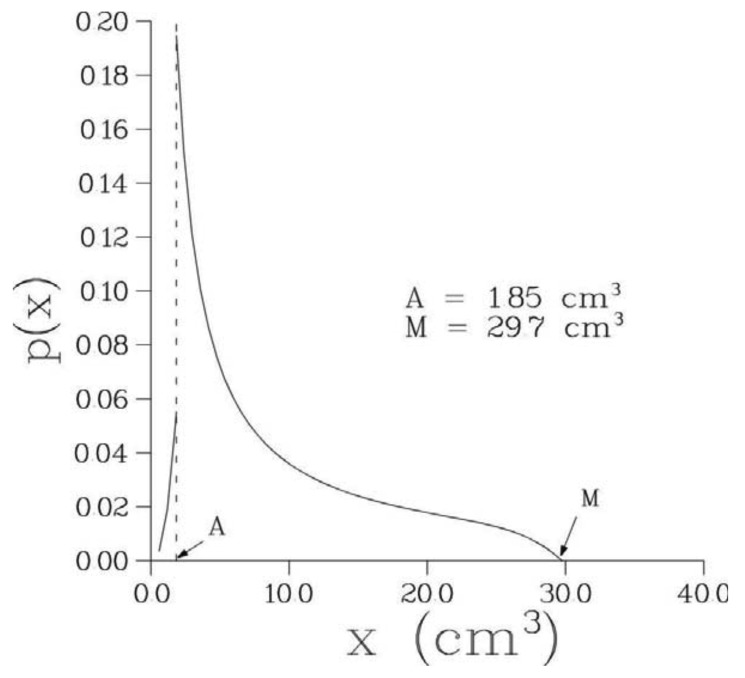
The probability density function, p(x), underlying the distribution of the volumes of detectable bone metastases, see Equations (13)-(16), for patient 1. Note the jump discontinuity at point A = 1.85 cm^3^ where p(A+)/p(A-) = γ_1_/γ_0_ = 3.5. Parameter A represents the volume of a metastasis whose inception occurred at the time of surgery while M is the maximum possible volume of bone metastases, that is, the volume of a metastasis whose inception occurred at onset of the primary tumor.

Optimal parameter values of the 6-parametric Full model along with the minimal values of –(log L)/n, where n is the number of bone metastases observed in a given patient, are presented in Table 2 while optimal values of biological parameters are given in Table 3. The optimized model provided an excellent fit to the empirical distribution of the volumes of detectable metastases for all patients; see Figure 2 where empirical and theoretical distribution functions are presented for one particular patient. The corresponding pdf, p(x), for this patient is shown in Figure 3.

For all patients, the optimal value of parameter A was equal to (more exactly, approached from the right) one of the observed volumes of metastases. The values of parameter θ were small for all patients. Therefore, we also applied the corresponding 4-parametric Homogeneous model (θ = 0), see Section 5. For all patients, the estimates of biological parameters γ_0_, γ_1_, ρ and T of the Homogeneous model and the likelihood agree reasonably well with (and for patients 3 and 9 are very close to) those for the Full model, see Tables 3 and 4. Along with the age at onset T computed through Equation (25), Tables 3 and 4 also give the age at inception of the first bone metastasis (which, according to the model, is the metastasis with the largest observed volume). At that age, primary tumors of all patients were extremely small so that application of any deterministic law of tumor growth for their estimation would be misleading.

As a self-consistency check, we applied the non-surgery model with two growth rates of the primary tumor (β_0_ before and β_1_ after the start of treatment) to the two surgery patients (patients 1, 2). As expected, the estimated values of β_1_ were small negative numbers: β_1_ = −2.3×10^7^ year^−1^ for patient 1 and β_1_ = −60.4 year^−1^ for patient 2.

## 9. Summary of Results

The results presented in Tables 2-4 lead to the following tentative conclusions about the natural history of metastatic prostate cancer and the effects of its treatment, in good agreement with a similar analysis of a breast cancer patient [55], see Section 2.

### 9.1. Onset of the Primary Tumor

The age at cancer onset displayed a substantial inter-patient variability. For several patients, the disease started in the early childhood, for others in the early middle ages, and for the rest of the patients, in their 50s, 60s or 70s. Such variation can be understood in terms of the heterogeneity of the disease: for some patients, the disease may be heritable or resulting from critical mutations occurring during gestation or early childhood while for others, the initiating genomic events could have occurred later or involved a long promotion time between the first genomic event and the emergence of the first malignant cell. The possibility of very early onset of adult cancers is well recognized. As suggested in [61,62], the earlier the occurrence of critical mutations leading to a particular cancer the larger is the number of stem cells carrying these mutations and hence also the risk of early cancer onset. Further evidence for the possibility of early onset of prostate cancer comes from the good agreement between the estimates of the age T at cancer onset provided by the Full and Homogeneous models for the majority of patients, see Tables 3 and 4 and note that T is an independent biological parameter of the Homogeneous model while within the Full model it is computed through Equation (25) on the basis of other model parameters.

### 9.2. Heterogeneity of Tumor Growth Rates

The pre-treatment rate of growth of the primary tumor displayed substantial variability among the 12 cancer patients analyzed. As shown in Table 3, these rates varied between 0.4 and 34.1 year^−1^ (equivalently, the tumor doubling times varied between 7.4 and 632.5 days). The rates of growth of metastases before and after the start of treatment also varied widely between the patients. Finally, for 9 out of 12 patients, the rate of growth of metastases after the start of treatment exceeded the pre-treatment growth rate of the primary.

### 9.3. Effects of the Systemic Treatment on the Primary Tumor

Among 10 patients who received systemic treatment, 7 patients responded to the treatment with a very fast reduction in the size of the primary tumor. The response of patient 6 was much slower: after the start of treatment the volume shrinkage half-life of his primary tumor was about 149 days. Finally, for patients 10 and 12, the half-life of their primary tumor reduction was 6.9 and 7.7 years, respectively. Thus, according to the model, systemic treatment of their primary tumors essentially failed.

### 9.4. Metastasis Shedding

**Figure 4.**
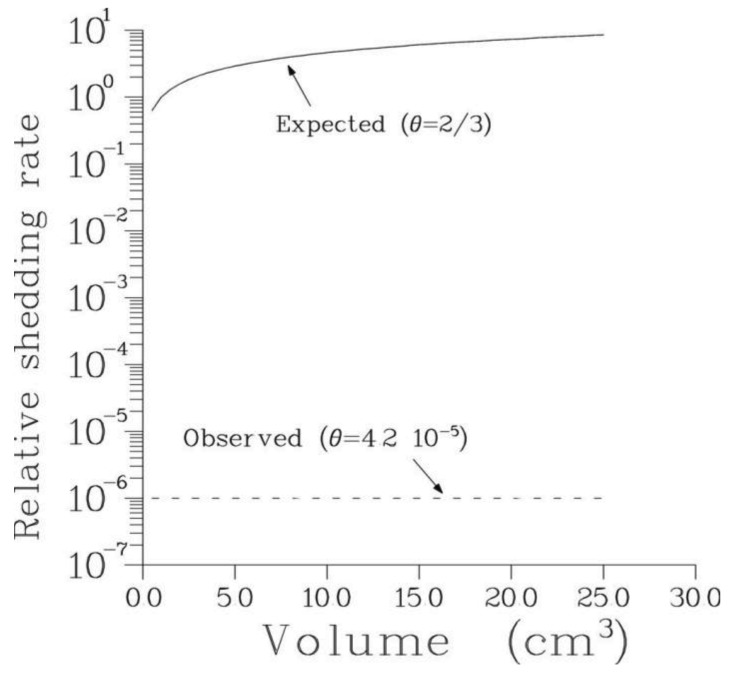
The rate of metastasis shedding Φ^θ^ for patient 1 (assuming α =10^−6^, see Equation (1)) as a function of primary tumor volume v corresponding to the tumor size Φ (v = Φ c, where c = 10^−9^ cm^3^ is taken to represent the average volume of a single tumor cell). The curve labeled “expected (θ = 2/3)” refers to the plausible (yet incorrect) assumption that metastases are generated by actively proliferating cancer cells localized at the tumor boundary. The curve labeled “observed (θ = 4.2×10^−5^)” describes the shedding rate for the value of θ estimated from the data. This, essentially constant, shedding rate implies that, contrary to the traditional belief, treatment of the primary is unlikely to substantially reduce the probability of metastatic failure. The log-plots of Φ^θ^ as functions of time rather than volume for the same values of θ are represented by two lines: one with the slope 2β_0_/3 and the other essentially horizontal. As discussed in the text, this supports the notion of prostate cancer stem cells.

According to Equation (1) the rate of metastasis shedding depends critically on the value of parameter θ. The latter was found to be uniformly small for all patients analyzed. Thus, the rate of metastasis shedding is essentially constant in time as long as primary tumor remains *in situ*. This also implies that treatment of the primary tumor has only weak effect on the rate of metastasis shedding and consequently on the total number of viable secondary tumors. This is illustrated in Figure 4.

### 9.5. Onset of Metastasis

**Figure 5.**
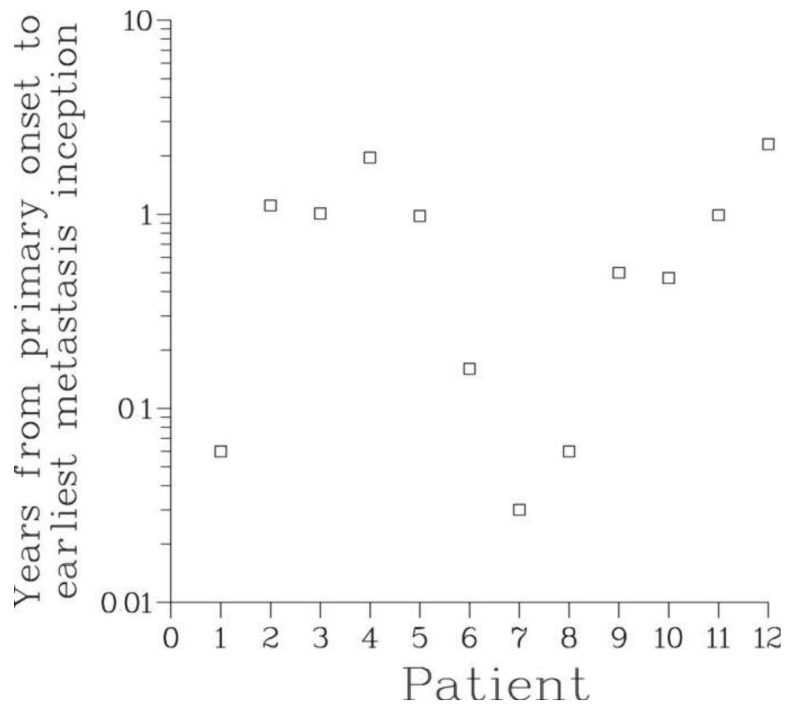
The earliest metastasis inception appears to occur within a relatively short time (2.3 years or less) following the onset of the primary tumor.

Our analysis confirms unequivocally that in all patients metastatic dissemination of prostate cancer occurred soon after the onset of the disease and much earlier than the appearance of a clinically detectable primary tumor. In fact, according to the Full model, the time between the onset of the disease and inception of the first metastasis never exceeded 2.3 years, see Table 3 and Figure 5. At that time, the primary tumor was extremely small and certainly undetectable. Shedding of the first viable metastasis occurred even earlier. Thus, for all patients, their disease was systemic at the outset, in agreement with hypothesis A1.

### 9.6. Metastasis Latency

**Figure 6.**
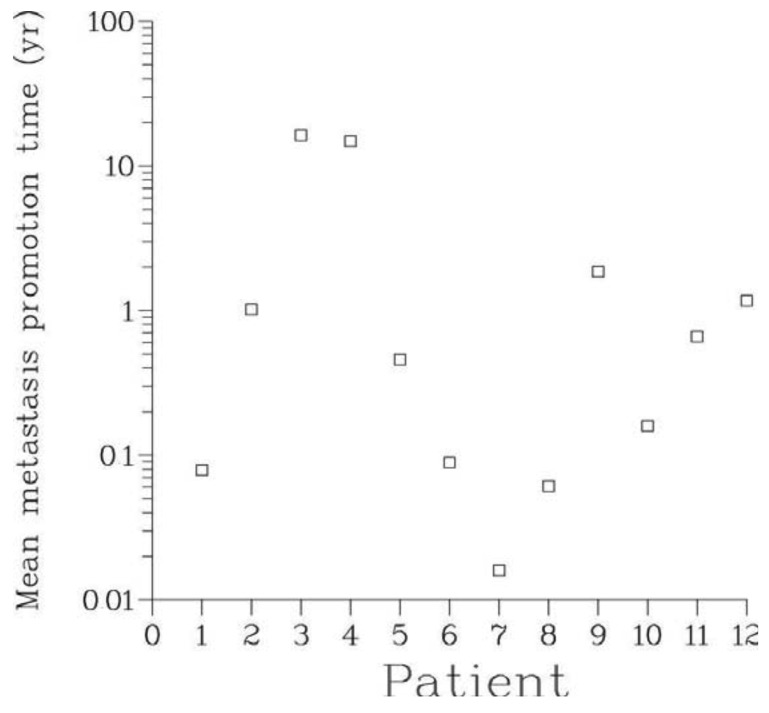
Expected metastasis latency times, ρ, in patients 1-12. ρ represents the average time spent by a viable metastasis between detachment from the primary tumor and onset of irreversible proliferation in a given secondary site (here, bone).

The mean metastasis latency times ρ computed through the Full model ranged from a few days to as long as 16.3 years, see Table 3 and Figure 6. This supports the notion of metastasis dormancy (hypothesis A2). For patients with long latency times, many metastases were still occult at the time of surveying. It can be hypothesized that the significant inter-patient variability of the latency times is due to heterogeneity in the genetic make-up of cancer cells and conditions of host microenvironment.

### 9.7. Inception of Metastases

Importantly, parameter A, see Equation (12), represents the size at age W of a metastasis whose inception occurred at the start of treatment (see Figure 1). Comparison of the values of this parameter with the observed volumes of metastases shows that in all patients inception of all or most of the detected metastases occurred prior to the start of treatment. Additionally, these early metastases have the largest volumes. Therefore, resection or irradiation of the primary tumor (or any other form of local treatment) has only minor effect on the number of metastases relevant for patients' survival.

### 9.8. Treatment-induced Acceleration of Metastatic Growth

**Figure 7.**
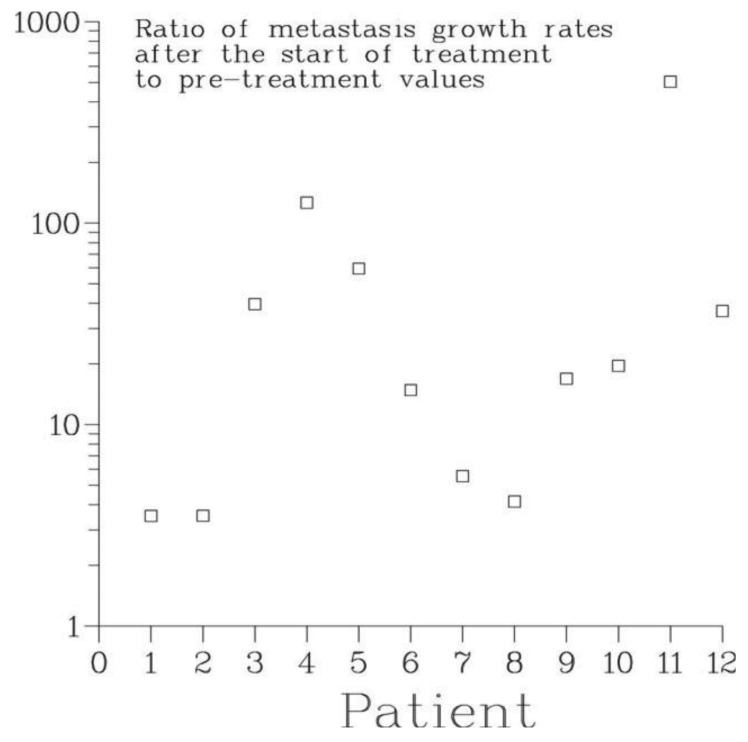
The ratios γ_1_/γ_0_ of the rates of growth of bone metastases after the start of treatment and before treatment for patients 1-12 are all significantly larger than 1. In case of chemotherapy, this unexpected feature may be the result of a treatment-related selection of most resistant and fastest growing metastases while in the case of surgery and possibly radiation it is likely due to the treatment-induced acceleration of the growth and vascularization of dormant or slowly growing secondary tumors, see Sections 1 and 9.

The effect of treatment of the primary on metastatic growth is characterized by the ratio γ_1_/γ_0_ of the rate of growth of bone metastases after the start of treatment to their pre-treatment growth rate. For all patients, this ratio was larger than 1, see Figure 7. According to the Full model, the smallest one, 3.5, occurred for patient 1 whose treatment consisted of surgery, external beam radiation and chemotherapy while the largest, 504, occurred for patient 11 who received chemo- and hormonal therapies alone. For patient 2, the second patient who had surgery and chemotherapy, the value of γ_1_/γ_0_ was 35.3. For all other patients, the ratio varied between 4 and 126. Thus, all modes of treatment of the primary lead to a dramatic irreversible exacerbation of the disease. At the same time, this suggests that primary tumor *in situ* has a strong inhibiting effect on the growth of secondary tumors. In fact, for all patients analyzed, the pre-treatment rate of growth of metastases was smaller than the rate of growth of the primary by an order of magnitude, see Table 3.

**Figure 8.**
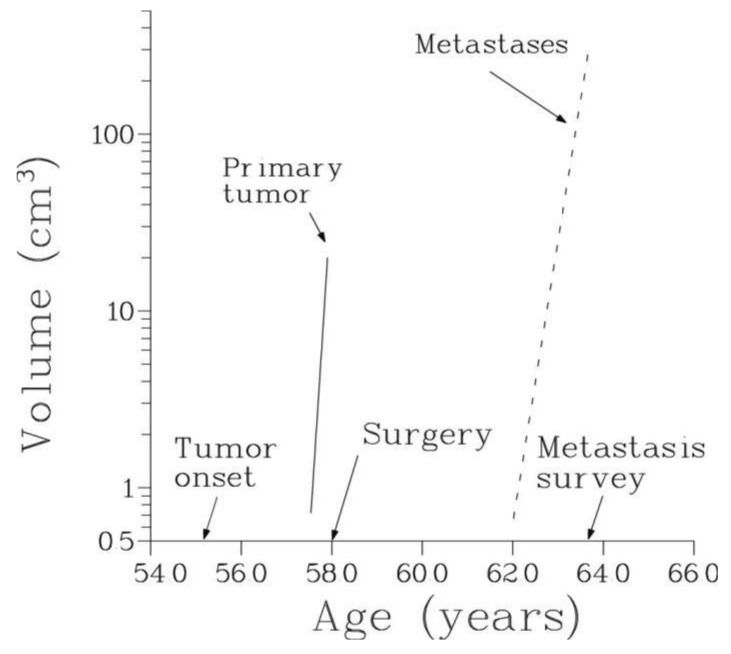
The volume of the primary tumor and the total volume of all detectable metastases represented as functions of age for patient 1. It is notable that as time progresses, the total metastatic volume by far exceeds the volume of the primary at the time of surgery. The total volume of metastases is dominated by the volume of the largest metastasis (that is, the metastasis with the earliest inception time, see also Figure 5).

As discussed in Section 3, the mechanism is likely to involve treatment-related weakening or total abrogation of the metastasis-inhibiting action of the primary tumor, as well as direct accelerating impact of treatment on metastatic growth. For surgery and radiation, the latter is caused by local and systemic production of growth and angiogenesis factors due to wound healing processes while in the case of chemo- and hormonal therapy it may be due to selection of fastest growing and most resistant cells. Finally, it is well-known [28] that avascular tumors cannot reach sizes exceeding a few mm in diameter. This suggests that at the start of treatment bone metastases in all patients were avascular, while at the time of their detection they reached considerable sizes unattainable for avascular tumors. Therefore, it is very likely that one of the most notable effects of treatment is turning on the angiogenic switch. The question as to how chemo- and/or hormonal therapies bring about this effect calls for further study. Thus, our analysis unequivocally supports hypotheses B1 and B2. Finally, accelerated growth of metastases after the start of treatment brings about a sharp increase in the total metastatic burden; see Figure 8, where the total volume of all detectable metastases is compared to the volume of the primary tumor.

## 10. Discussion and Conclusions

The field of oncology has so far been dominated by the tacit notion that treating the primary tumor reduces the chance of distant metastatic failure and is thereby beneficial with respect to the patient's life expectancy. This belief is based on four premises. First, the probability of developing metastases increases in direct relation to the volume of the primary tumor. Second, the longer the primary tumor is *in situ*, the larger the number of metastases it produces. Third, the number of metastases at presentation is insignificant, *i.e.*, the onset of metastasis is typically a late event in relation to the development of a clinically observable primary tumor. Fourth, it is taken as axiomatic that treatment of the primary – even if suboptimal in terms of controlling the distant spread of the disease – does not cause a detrimental outcome. If correct, the *sine qua non* of the above notion is that the earlier and more aggressive the treatment the better the outcome.

Our results directly challenge the validity of these premises. It follows from Equations (9) and (10) that the probability of developing metastases and their expected number increase with the time the primary tumor is *in situ*. However, since θ is invariably very small, this dependence is not critical. For example, in our analysis the expected number of viable metastases grows essentially linearly in time. Equally important, the third premise appears to be in error: by the time of primary tumor diagnosis there are already a large number of viable metastases dormant or slowly growing at various secondary sites, which makes the reduction of the number of subsequently formed secondary tumors resulting from the treatment of the primary tumor practically irrelevant. Finally, and most importantly, based on our (admittedly limited) analysis, surgery and chemotherapy of metastatic prostate cancer accelerate the progression of the disease.

While in the case of surgery the mechanisms underlying this effect have been known for decades and are fairly well understood, this is not the case for systemic treatment. A number of important questions arise and call for further study: (1) what is the role of selection of resistant and/or fast proliferating cancer cells in the sharp increase in the average rate of growth of metastases predicted by the model? (2) Does systemic treatment cause a drop in the number of metastases or only elimination of slowly growing cells within each metastasis followed by its repopulation by fast growing resistant cells? (3) Does elimination of metastases or reduction in their size have the same boosting effect on other metastases as would the elimination of the primary tumor?

If confirmed, the results of this work potentially could have profound effects on the strategies of cancer treatment. They would place more emphasis on the control of cancer progression through maintaining the state of homeostasis including dormancy, in particular by encouraging watchful waiting. Although such conservative strategies carry significant risks of their own, they should be balanced against the impending risks of aggressive treatment demonstrated in this work.

## Figures and Tables

**Table 1.** Descriptive characteristics of patient cohort (NA=not available; SX=surgery; RX = radiation therapy).

**No.**	**Pre-treatment PSA/Gleason Score**	**SX/RX**	**Systemic treatment only (Y/N)**	**Age at treatment(year)**	**Age at PET/CT study (year)**	**Primary tumor volume** † **(cm^3^)**	**Number of metastatic lesions**	**Volume of the largest metastasis (cm^3^)**

1	6.6/9	SX/RX	N	57.9	63.7	27	36	28
2	NA/7	SX	N	50.8	64.6	(20)	22	14
3	1365/9	-	Y	75.7	80.1	(20)	45	37
4	124/9	-	Y	48.1	50.7	19.1	30	37
5	19.8/5	-	Y	56.5	63.2	26.6	22	36
6	33/7	-	Y	60.6	62.1	(20)	24	19
7	856/9	-	Y	74.3	75.1	(20)	58	68
8	284/8	-	Y	66.8	69.6	(20)	32	47
9	24/8	-	Y	70.9	77.0	(20)	27	28
10	60/8	-	Y	63.6	71.8	(20)	10	33
11	7.3/7	-	Y	71.8	72.7	47	22	21
12	46/6	-	Y	57.3	66.0	(20)	18	35

† A primary tumor volume of 20 cm^3^ (indicated in parentheses) was assigned when information on this quantity was unavailable. Quantities derived from the primary tumor volume, see Equation (27), depend on the logarithm of S and, as a result, vary only slightly with S.

**Table 2.** Optimal Parameters of the Full model.

**Patient No**	**a_0_**	**a_1_**	**b_0_**	**b_1_**	**A (cm^3^)**	**M (cm^3^)**

1	3.62×10^−4^	-	12.00	3.41	1.85	29.74
2	3.45×10^−6^	-	22.50	0.64	1.80	15.09
3	2.88×10^−6^	-5.03	0.49	0.01	2.46	42.00
4	2.37×10^−3^	-3.89	1.02	8.07×10^−3^	2.34	41.89
5	2.36×10^−5^	-4.78	39.67	0.67	2.55	38.18
6	1.06	-2.81	11.95	0.80	2.15	22.67
7	0.56	-18.00	12.90	2.33	2.95	76.30
8	0.64	-4.33	8.41	2.03	4.63	52.67
9	8.81×10^−5^	-8.39	2.55	0.15	2.49	30.68
10	1.14	-1.16	43.05	2.20	10.34	35.78
11	1.18×10^−3^	-11.52	31.47	6.25×10^−2^	2.73	22.45
12	0.53	-3.73	12.92	0.35	1.24	40.76

**Table 3.** Biological parameters of the full model.

**No.**	**γ_0_** **(year^−1^)**	**γ_1_** **(year^−1^)**	**γ_1_/γ_0_**	**β_0_** **(year^−1^)**	**β1** **(year^−1^)**	**ρ** **(year)**	**θ**	**Age at tumor onset** **(year)**	**Age at the earliest metastatic inception** **(year)**	**−(logL)/n**

1	1.047	3.684	3.5	9.1		0.08	4.2×10^−5^	55.3	55.3	3.04
2	0.044	1.543	35.3	0.5		1.0	3.1×10^−7^	2.2	3.3	2.39
3	0.120	4.971	39.7	1.0	−4.6×10^4^	16.3	3.4×10^−7^	53.1	54.1	2.91
4	0.066	8.362	126.4	0.5	−7.1	14.8	2.9×10^−4^	4.5	6.5	2.95
5	0.054	3.238	59.5	0.5	−1.6×10^3^	0.46	2.7×10^−6^	6.8	7.8	3.29
6	0.930	13.864	14.8	9.4	−1.7	0.090	0.10	58.1	58.2	2.27
7	4.680	25.958	5.5	34.1	−198.4	0.016	7.6×10^−2^	73.6	73	3.28
8	1.940	8.034	4.1	18.9	−30.7	0.061	6.6×10^−2^	65.6	65.6	3.34
9	0.210	3.570	16.9	2.0	−l.1×10^4^	1.9	9.3×10^−6^	59.0	59.5	3.04
10	0.140	2.805	19.6	2.7	−0.1	0.16	6.0×10^−2^	55.0	55.4	3.23
11	0.048	24.142	503.7	0.6	−10.9	0.66	1.0×10^−4^	27.8	28.8	3.01
12	0.066	2.412	36.6	0.4	−0.09	1.17	7.8×10^−2^	4.3	6.6	2.58

**Table 4.** Biological parameters of the Homogeneous model (θ = 0).

**No.**	**γ_0_** **(year**^−^**^1^)**	**γ_1_** **(year**^−^**^1^)**	**γ_1_/γ_0_**	**ρ** **(year)**	**Age T at tumor onset** **(year)**	**Age at the earliest metastatic inception** **(year)**	**−(logL)/n**

1	2.72	3.70	1.4	0.06	56.9	56.9	3.05
2	0.045	1.54	34.2	6.8	2.3	4.7	2.42
3	0.11	4.97	43.9	15.5	50.8	51.8	2.91
4	0.096	8.36	87.5	10.1	17.9	19.3	2.96
5	0.049	3.24	65.9	1.8	0.7	2.5	3.31
6	0.63	13.73	21.9	7.6	56.5	56.8	2.32
7	0.08	25.50	332.1	21.3	27.7	28.5	3.30
8	0.44	7.80	17.8	1.1	59.7	60.0	3.43
9	0.18	3.57	19.9	2.2	56.9	57.5	3.04
10	0.10	2.68	25.1	2.3	42.0	43.0	3.58
11	0.042	24.14	580.7	2.2	19.0	22.2	3.02
12	0.069	2.41	34.7	33.8	6.0	9.0	2.60

## References

[1] Baum M., Chaplain M., Anderson A., Douek M., Vaidya J.S. (1999). Does breast cancer exist in a state of chaos?. Eur. J. Cancer.

[2] Demicheli R., Retsky M., Hrushesky W.J.M, Baum M., Gukas I.D. (2008). The effects of surgery on tumor growth: a century of investigations. Ann. Oncol..

[3] Retsky M., Demicheli R., Hrushesky W., Baum M., Gukas I. (2010). Surgery triggers outgrowth of latent distant disease in breast cancer: An inconvenient truth?. Cancers.

[4] Hanin L. (2010). Why victory in the war on cancer remains elusive: Biomedical hypotheses and mathematical models. Cancers.

[5] Bartoszyński R., Edler L., Hanin L., Kopp-Schneider A., Pavlova L., Tsodikov A., Zorin A., Yakovlev A. (2001). Modeling cancer detection: Tumor size as a source of information on unobservable stages of carcinogenesis. Math. Biosci..

[6] Hanin L.G., Rose J., Zaider M. (2006). A stochastic model for the sizes of detectable metastases. J. Theor. Biol..

[7] Hanin L.G., Tan W.Y., Hanin L.G. (2008). Distribution of the sizes of metastases: mathematical and biomedical considerations. Handbook of Cancer Models with Applications.

[8] Douglas J.R.S. (1971). Significance of the size distribution of bloodborne metastases. Cancer.

[9] Fisher B. (1980). Laboratory and clinical research in breast cancer: a personal adventure. The David A. Karnofsky memorial lecture. Cancer Res..

[10] Barbour A., Gotley D.C. (2003). Current concepts of tumour metastasis. Ann. Acad. Med. Singapore.

[11] Ashworth T.R. (1869). A case of cancer in which cells similar to those in the tumour were seen in the blood after death. Med. J. Australia.

[12] Goldmann E.E. (1897). Anatomische Untersuchungen über die Verbreitungswege bösartiger Geschwülstle. Beiträge zur Klinischen Chirurgie.

[13] Fodstad O., Faye R., Hoifodt H.K., Skovlund E., Aamdal S. (2001). Immunobead-based detection and characterization of circulating tumor cells in melanoma patients. Recent Results Cancer Res..

[14] Pantel K., Otte M. (2001). Occult micrometastases: enrichment, identification and characterization of single disseminated tumour cells. Semin. Cancer Biol..

[15] Jiao X., Krasna M.J. (2002). Clinical significance of micrometastasis in lung and esophageal cancer: a new paradigm in thoracic oncology. Ann. Thorac. Surg..

[16] Sugio K., Kase S., Sakada T., Yamazaki K., Yamaguchi M., Ondo K., Yano T. (2002). Micrometastasis in the bone marrow of patients with lung cancer associated with a reduced expression of E-cadherin and beta-catenin: risk assessment by immunohistochemistry. Surgery.

[17] Paget S. (1889). The distribution of secondary growths in cancer of the breast. Lancet.

[18] Hadfield G. (1954). The dormant cancer cell. Br. Med. J..

[19] Sugarbaker E.V., Ketcham A.S., Cohen A.M. (1971). Studies of dormant tumor cells. Cancer.

[20] Meng S., Tripathy D., Frenkel E.P., Shete S., Naftalis E.Z., Huth J.F., Beitsch P.D., Leitch M., Hoover S., Euhus D., Haley B., Morrison L., Fleming T.P., Herlyn D., Terstappen L.W.M.M., Fehm T., Tucker T.F., Lane N., Wang J., Uhr J.W. (2004). Circulating tumour cells in patients with breast cancer dormancy. Clin. Cancer Res..

[21] Luzzi K.J., MacDonald I.C., Schmidt E.E., Kerkvliet N., Morris V.L., Chambers A.F., Groom A.C. (1998). Multistep nature of metastatic inefficiency. Dormancy of solitary cells after successful extravasation and limited survival of early micrometastases. Am. J. Pathol..

[22] Naumov G.N., MacDonald I.C., Weinmeister P.M., Kerkvliet N., Nadkarni K.V., Wilson S.M., Morris V.L., Groom A.C., Chambers A.F. (2002). Persistence of solitary mammary carcinoma cells in a secondary site: a possible contributor to dormancy. Cancer Res..

[23] Vessella R.L., Pantel K., Mohla S. (2007). Tumor cell dormancy. An NCI Workshop Report. Cancer Biol.Ther..

[24] Marches R., Scheuermann R., Uhr J. (2006). Cancer dormancy: From mice to man. Cell Cycle.

[25] Ellis W.J., Pfitzenmaier J., Colli J., Arfman E., Lange P.H., Vessella R.L. (2003). Detection and isolation of prostate cancer cells from peripheral blood and bone marrow. Urology.

[26] Pfitzenmaier J., Vessella R.L., Ellis W.J., Lange P.H., Pantel K. (2003). Detection, isolation and study of disseminated prostate cancer cells in the peripheral blood and bone marrow. Micrometastasis.

[27] Karrison T.G., Ferguson D.J., Meier P. (1999). Dormancy of mammary carcinoma after mastectomy. J. Natl. Cancer Inst..

[28] Folkman J., Watson K., Ingber D., Hanahan D. (1989). Induction of angiogenesis during the transition from hyperplasia to neoplasia. Nature.

[29] Demicheli R. (2001). Tumour dormancy: Findings and hypotheses from clinical research on breast cancer. Semin. Cancer Biol..

[30] Bonnet D., Dick J.E. (1997). Human acute myeloid leukemia is organized as a hierarchy that originates from a primitive hematopoietic cell. Nature Medicine.

[31] Collins A.T., Berry P.A., Hyde C., Stower M.J., Maitland N.J. (2005). Prospective identification of tumorigenic prostate cancer stem cells. Cancer Res..

[32] Ehrlich P., Apolant H. (1905). Beobachtungen über maligne Mäusetumoren. Berliner Klinische Wochenschrift.

[33] Bashford E., Murray J.A., Cramer W. (1907). The natural and induced resistance of mice to the growth of cancer. Proc. Royal Soc. London.

[34] Marie P., Clunet J. (1910). Fréquences des métastases viscérales chez les souris cancéreuses après ablation chirurgicale de leur tumeur. Bulletin Association Française pour L'étude du Cancér.

[35] Tyzzer E.E. (1913). Factors in the production and growth of tumor metastases. J. Med. Res..

[36] Simpson-Herren L., Sanford A.H., Holmquist J.P. (1976). Effects of surgery on the cell kinetics of residual tumor. Cancer Treat. Rep..

[37] Sugarbaker E., Thornthwaite J., Ketcham A., Day S.B., Myers W.P.L., Stanley P., Garattini S., Lewis M.G. (1977). Inhibitory effect of a primary tumor on metastasis. Progress in Cancer Research and Therapy.

[38] Fischer B., Gunduz N., Saffer E. (1983). Influence of the interval between primary tumor removal and chemotherapy on kinetics and growth of metastases. Cancer Res..

[39] Lange P.H., Hekmat K., Bosl G., Kennedy B.J., Fraley E.E. (1980). Accelerated growth of testicular cancer after cytoreductive surgery. Cancer.

[40] De Giorgi V., Massi D., Gerlini G., Mannone F., Quercioli E., Carli P. (2003). Immediate local and regional recurrence after the excision of a polypoid melanoma: Tumor dormancy or tumor activation?. Dermatologic Surgery.

[41] Tseng W.W., Doyle J.A., Maguiness S., Horvai A.E., Kashani-Sabet M., Leong S.P.L. (2009). Giant cutaneous melanomas: evidence for primary tumour induced dormancy in metastatic sites?. Br. Med. J. Case Reports.

[42] Sandler H.M., Hanks G.E. (1988). Analysis of the possibility that transurethral resection promotes metastasis in prostate cancer. Cancer.

[43] Mitsudomi T., Nishioka K., Maruyama R., Saitoh G., Hamatake M., Fukuyama Y., Yaita H., Ishida T., Sugimachi K. (1996). Kinetic analysis of recurrence and survival after potentially curative resection of nonsmall cell lung cancer. J. Surg. Oncol..

[44] Smolle J., Soyer H.P., Smolle-Juttner F.M., Rieger E., Kerl H. (1997). Does surgical removal of primary melanoma trigger growth of occult metastases? An analytical epidemiological approach. Dermatologic Surgery.

[45] Demicheli R., Retsky M.W., Swartzendruber D.E., Bonadonna G. (1997). Proposal for a new model of breast cancer metastatic development. Ann. Oncol..

[46] Retsky M.W., Demicheli R., Swartzendruber D.E., Bame P.D., Wardwell R.H., Bonadonna G., Speer J., Valagussa P. (1997). Computer simulation of a breast cancer metastasis model. Breast Cancer Res. Treat..

[47] O'Reilly M.S., Holmgren L., Shing Y., Chen C., Rosenthal R.A., Moses M., Lane W.S., Cao Y., Sage E.H., Folkman J. (1994). Angiostatin: A novel angiogenesis inhibitor that mediates the suppression of metastases by a Lewis lung carcinoma. Cell.

[48] Retsky M., Demicheli R., Hrushesky W. (2003). Breast cancer screening: Controversies and future directions. Curr. Opin. Obstet. Gynecol..

[49] Retsky M., Bonadonna G., Demicheli R., Folkman J., Hrushesky W., Valagussa P. (2004). Hypothesis: Induced angiogenesis after surgery in premenopausal node-positive breast cancer patients is a major underlying reason why adjuvant chemotherapy works particularly well for those patients. Breast Cancer.

[50] Lacy A.M., Garcia-Valdecasas J.C., Delgado S., Castells A., Taurá P., Piqué J.M., Visa J. (2002). Laporoscopy-assisted colectomy versus open colectomy for treatment of non-metastatic colon cancer: a randomized trial. Lancet.

[51] Hoover H.C., Ketcham A.S. (1975). Techniques for inhibiting tumor metastases. Cancer.

[52] Prehn R.T. (1993). Two competing influences that may explain concomitant tumor resistance. Cancer Res..

[53] Maida V., Ennis M., Kuziemsky C., Corban J. (2009). Wounds and survival in cancer patients. Eur. J. Cancer.

[54] Kendal W.S. (2005). Chance mechanisms affecting the burden of metastases. BMC Cancer.

[55] Hanin L.G., Korosteleva O. (2010). Does extirpation of the primary breast tumor give boost to growth of metastases? Evidence revealed by mathematical modeling. Math. Biosci..

[56] Chambers A.F., Macdonald I.F., Schmidt E., Koop S., Morris V.L., Khokha R., Groom A.C. (1995). Steps in tumor metastasis: new concepts from intravital videomicroscopy. Cancer Metastasis Rev..

[57] Fidler I.J., DeVita V.T., Hellman S., Rosenberg S.A. (1997). Molecular biology of cancer: invasion and metastasis. Cancer Principles and Practice of Oncology.

[58] Ross S.M. (1997). Introduction to Probability Models.

[59] Hanin L.G., Yakovlev A.Y. (1996). A nonidentifiability aspect of the two-stage model of carcinogenesis. Risk Analysis.

[60] Hanin L.G. (2002). Identification problem for stochastic models with application to carcinogenesis, cancer detection and radiation biology. Discrete Dynamics in Nature and Society.

[61] Frank S.A., Nowak M.A. (2003). Developmental predisposition to cancer. Nature.

[62] Meza R., Luebeck E.G., Moolgavkar S.H. (2005). Gestational mutations and carcinogenesis. Math. Biosci..

